# Central venous O_2 _saturation and venous-to-arterial CO_2 _difference as complementary tools for goal-directed therapy during high-risk surgery

**DOI:** 10.1186/cc9310

**Published:** 2010-10-29

**Authors:** Emmanuel Futier, Emmanuel Robin, Matthieu Jabaudon, Renaud Guerin, Antoine Petit, Jean-Etienne Bazin, Jean-Michel Constantin, Benoit Vallet

**Affiliations:** 1 Department of Anaesthesiology and Critical Care Medicine, Estaing Hospital, University Hospital of Clermont-Ferrand, 1 Place Lucie Aubrac, Clermont-Ferrand, 63000, France; 2 Federation of Anaesthesiology and Critical Care Medicine, University Hospital of Lille, Univ Nord de France, Rue du Pr. Emile Laine, Lille, 59037, France

## Abstract

**Introduction:**

Central venous oxygen saturation (ScvO_2_) is a useful therapeutic target in septic shock and high-risk surgery. We tested the hypothesis that central venous-to-arterial carbon dioxide difference (P(cv-a)CO_2_), a global index of tissue perfusion, could be used as a complementary tool to ScvO_2 _for goal-directed fluid therapy (GDT) to identify persistent low flow after optimization of preload has been achieved by fluid loading during high-risk surgery.

**Methods:**

This is a secondary analysis of results obtained in a study involving 70 adult patients (ASA I to III), undergoing major abdominal surgery, and treated with an individualized goal-directed fluid replacement therapy. All patients were managed to maintain a respiratory variation in peak aortic flow velocity below 13%. Cardiac index (CI), oxygen delivery index (DO_2_i), ScvO_2_, P(cv-a)CO_2 _and postoperative complications were recorded blindly for all patients.

**Results:**

A total of 34% of patients developed postoperative complications. At baseline, there was no difference in demographic or haemodynamic variables between patients who developed complications and those who did not. In patients with complications, during surgery, both mean ScvO_2 _(78 ± 4 versus 81 ± 4%, *P *= 0.017) and minimal ScvO_2 _(minScvO_2_) (67 ± 6 versus 72 ± 6%, *P *= 0.0017) were lower than in patients without complications, despite perfusion of similar volumes of fluids and comparable CI and DO_2_i values. The optimal ScvO_2 _cut-off value was 70.6% and minScvO_2 _< 70% was independently associated with the development of postoperative complications (OR = 4.2 (95% CI: 1.1 to 14.4), *P *= 0.025). P(cv-a)CO_2 _was larger in patients with complications (7.8 ± 2 versus 5.6 ± 2 mmHg, *P *< 10^-6^). In patients with complications and ScvO_2 _≥71%, P(cv-a)CO_2 _was also significantly larger (7.7 ± 2 versus 5.5 ± 2 mmHg, *P *< 10^-6^) than in patients without complications. The area under the receiver operating characteristic (ROC) curve was 0.785 (95% CI: 0.74 to 0.83) for discrimination of patients with ScvO_2 _≥71% who did and did not develop complications, with 5 mmHg as the most predictive threshold value.

**Conclusions:**

ScvO_2 _reflects important changes in O_2 _delivery in relation to O_2 _needs during the perioperative period. A P(cv-a)CO_2 _< 5 mmHg might serve as a complementary target to ScvO_2 _during GDT to identify persistent inadequacy of the circulatory response in face of metabolic requirements when an ScvO_2 _≥71% is achieved.

**Trial registration:**

Clinicaltrials.gov Identifier: NCT00852449.

## Introduction

Adequate tissue perfusion is an essential component of oxygenation during high-risk surgery and may improve outcome [1,2]. Careful monitoring of fluid administration by individualized goal-directed therapy (GDT) has been shown to reduce organ failure and hospital stay [3-5]. As a supplement to routine cardiovascular monitoring, GDT aims to optimize O_2 _delivery (DO_2_) through defined goals, based on maximization of flow-related haemodynamic parameters [6-10], while avoiding hypovolaemia and fluid overload which may alter tissue oxygenation [11,12].

In addition, the use of early warning signals of tissue hypoxia, such as central venous oxygen saturation (ScvO_2_), which reflects important changes in the O_2 _delivery/consumption (DO_2_/VO_2_) relationship, has been found to be useful during high-risk surgery [13-15]. Indeed, previous studies have shown that changes in ScvO_2 _closely reflect circulatory disturbances during periods of tissue hypoxia [16], and that low ScvO_2 _is associated with increased postoperative complications [13-15]. Furthermore, by closely monitoring of tissue O_2 _extraction, calculated from ScvO_2_, early correction of altered tissue oxygenation with appropriate fluid loading in conjunction with low doses of inotropes was found to reduce postoperative organ failure in patients with poor O_2 _utilization [13].

In a recent randomized study of patients treated with an individualized GDT protocol [17], we found that, despite optimization of preload with repeated fluid loading, excessive fluid restriction increased postoperative complications in parallel with reduced ScvO_2 _values [17]. The ScvO_2_threshold value for predicting complications (approximately 71%) was similar to those reported previously [14,15]. Significant ScvO_2 _fluctuations may occur during both surgery and sepsis, and high ScvO_2 _values do not necessarily reflect changes in DO_2 _and macrocirculatory adequacy [18,19], which may therefore limit the clinical relevance of ScvO_2 _in routine practice. Persistent tissue hypoperfusion with increased ScvO_2 _and O_2 _extraction defects might be related to microcirculatory and/or mitochondrial failure [19,20].

Interestingly, central venous-to-arterial PCO_2 _(Pcv-aCO_2_), with central venous PCO_2 _as a surrogate for mixed venous PCO_2 _[21], has recently been proposed as a useful tool for GDT in ICU-septic patients to identify persistent hypoperfusion when a ScvO_2 _> 70% has been reached [20]. Decreased tissue blood flow (ischemic hypoxia) represents the major determinant in increased P(v-a)CO_2 _[22], and P(v-a)CO_2 _could therefore be considered as an indicator of adequate venous blood flow to remove CO_2 _produced by peripheral tissues [23,24].

The results of a previous study, which included patients treated with intraoperative GDT [17], were used to investigate whether P(cv-a)CO_2 _is useful for discriminating patients at risk of developing postoperative complications. It was hypothesized that P(cv-a)CO_2 _may be a useful complementary tool when a threshold ScvO_2 _value has been reached by individualized GDT during major abdominal surgery.

## Materials and methods

### Patients

The study that provided data [17] used here was approved by our Institutional Review Board, and all patients provided written informed consent. Data were collected from eligible patients with an ASA score of I to III scheduled for surgery with an expected duration of > 60 minutes. Surgical procedures included colon/rectum resections, gastric resections, duodenopancreatectomy and hepatectomy. Exclusion criteria included: age < 18 years, body mass index > 35 kg m^-2^, pregnancy, chronic obstructive pulmonary disease with forced expiratory volume in 1 sec < 50%, emergency surgery, coagulopathy, sepsis or systemic inflammatory response syndrome [25], significant hepatic (prothrombin ratio < 50%, factor V < 50%) or renal failure (creatinine > 50% upper limit of normal value), and those in whom epidural analgesia was contraindicated.

### Study protocol

The protocol and design of the original study have been described in detail elsewhere [17]. Briefly, patients were randomly assigned by a concealed allocation approach (computer-generated codes), using opaque sealed envelopes containing the randomization schedule, to 6 mL kg^-1 ^h^-1^(restricted-GDT group) or 12 mL kg^-1 ^h^-1^(conventional-GDT group) of crystalloids (lactated Ringer's solution), reflecting current clinical practice for restricted (R-GDT group) and more conventional (C-GDT group) fluid administration [26]. Study investigators, but not anaesthesiologists, were blinded to treatment assignments. Immediately after induction of anaesthesia, an oesophageal Doppler probe (HemoSonic 100, Arrow International, Everett, MA, USA) was inserted and adjusted to obtain the highest velocity signal from the descending aorta. Respiratory variations in peak aortic flow velocity (deltaPV) were monitored as described previously [27,28], and stroke volume and cardiac output were recorded continuously. Additional fluid boluses of 250 mL hydroxethylstarch (HES 130/0.4, Voluven^®^; Fresenius-Kabi, Bad Hamburg, Germany) were given in order to maintain deltaPV below 13% [28]. The fluid challenge was repeated (up to 50 mL kg^-1^), if necessary, until deltaPV was corrected. In other cases (deltaPV < 13% and evidence of haemodynamic instability), a vasoactive/inotropic support (ephedrine chlorhydrate or dobutamine) could be added. Blood was transfused in order to maintain haemoglobin > 8 g dL^-1 ^in all patients, or > 10 g dL^-1 ^in patients with a history of coronary artery disease. Perioperative management was similar in all patients except for the basal rate of intraoperative crystalloids.

### Data collection and outcome measures

Preoperatively, patients were equipped with central venous (positioned with the tip within the superior vena cava) and arterial catheters. Arterial and central venous blood gas analyses were performed by intermittent blood sampling and co-oximetry (IL Synthesis, Instrumentation Laboratory^®^, Lexington, MA, USA) 10 minutes before surgery (baseline), hourly throughout surgery and until discharge from the post-acute care unit (PACU). This equipment was calibrated each hour, and routine quality control checks were performed. Anaesthesiologists were blinded to ScvO_2_and Pcv-aCO_2 _measurements during the course of surgery, which were, therefore, not used to guide clinical management at any stage of the study.

During surgery, the following parameters were recorded: electrocardiogram, pulse oximetry, invasive arterial pressure, cardiac output, oxygen delivery index (DO_2_i), the infused volume of crystalloids, HES, the need for packed red blood cells (PRBCs) and vasoactive/inotrope support, and urine output. Serum lactate, haemoglobin, creatinine, C-reactive protein (CRP), procalcitonin (PCT) and albumin levels were measured at PACU admission and during the 48 h following surgery. Minimal ScvO_2 _(minScvO_2_) was considered as the lowest value during the course of surgery.

Postoperative complications were recorded systematically and assessed according to previously defined criteria [6,29,30]. For the purpose of this study, and to assess the effect of abnormal perfusion on tissue oxygenation, we focused specifically on postoperative septic complications, which seem the most relevant clinically in the context of digestive surgery. Diagnosis of postoperative sepsis was based on international consensus guidelines [25]. Infection consisted of postoperative intraabdominal abscesses, wound infections, pneumonia and urinary tract infections. Cardiovascular (congestive heart failure, pulmonary embolism), postoperative haemorrhage and reintervention, neurological (confusion), renal failure and respiratory complications (pneumothorax and pulmonary embolism) complications were not included in the data analysis, except if associated with sepsis. The definition of the complications has been described in detail elsewhere [17]. Pre- and post-operative data, and post-operative complications were recorded by non-research staff blinded to the patient's allocation group. These were verified, in accordance with predefined criteria, by a member of the research team unaware of study group allocation. This process involved inspection of radiological investigations, laboratory data and clinical assessment.

### Statistical analysis

Data in tables are presented as means ± standard deviation (SD) when normally distributed, as medians (interquartile range) when not normally distributed, or as a percentage of the group from which they were derived for categorical data. The chi^2 ^test was used to compare qualitative data. Qualitative and quantitative data were compared using the Student's *t*-test or analysis of variance (ANOVA) when normally distributed (and variance were equivalent), or the Mann-Whitney U-test or Kruskal-Wallis H test in other circumstances. A multivariate analysis of variance (MANOVA) was used to explore longitudinal data. Multiple logistic regression was employed to identify independent risk factors for postoperative complications. The results of logistic regression are reported as adjusted odds ratios with 95% confidence intervals (CI). The robustness of the model was assessed using a Hosmer-Lemeshow Goodness-of-Fit-Test [31]. Receiver operator characteristic (ROC) curves were constructed to identify optimal cut-off values for outcome associations. The optimal cut-off was defined as the value associated with the highest sum of sensitivity and specificity (Youden's index). Analysis was performed using SEM software [32] and significance was set at *P *< 0.05.

## Results

**Figure 1:**
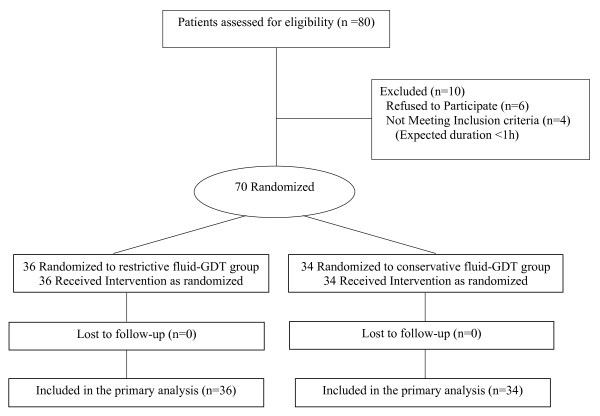
**Flow diagram of the original study**.

Complete follow-up data were collected from 70 patients included in the original study between May and December 2008 (Figure 1). Thirty patients developed postoperative complications (58% of the R-GDT group and 26% of the C-GDT group, *P *< 0.01), including 24 who developed at least one of the following: postoperative sepsis (*n *= 21), intra-abdominal abscess (*n *= 16), pneumonia (*n *= 7) and urinary tract infection (*n *= 4). There were six (8%) who had postoperative acute lung injuries or acute respiratory distress syndrome but none of them was associated with sepsis, and was, therefore, not included in the data analysis. There was no abdominal syndrome. There were two deaths (one in each group, *P *= 0.50). ScvO_2 _and P(cv-a)CO_2 _data were available for all patients. The demographics and commonly measured biological variables for the study participants are shown in Table 1. Surgical procedures consisted of colon/rectum resections (43%), duodenopancreatectomy (20%), gastrectomy (21%) and hepatectomy (16%), and were equally distributed (*P *= 0.87). There were no differences in operative time and blood loss between the two groups: 248 ± 42 *vs*. 233 ± 62 min (*P *= 0.21) and 326 ± 215 *vs*. 357 ± 373 ml (*P *= 0.68), respectively, in patients with and without complications. All patients were extubated within two hours after surgery.

**Table 1:** Demographic and biological data at inclusion for patients with and without postoperative complications

	Patients with complications (*n *= 24)	Patients without complications (*n *= 46)	*P*
**Demographic**			
Age (years)	60 ± 13	62 ± 13	0.61
Sex M/F (%)	62/38	52/48	0.41
BMI (kg m^-2^)	28 ± 7	25 ± 3	0.06
P-POSSUM score	35 ± 6.6	33 ± 5.6	0.21
ASA score I/II/III	12/63/25	11/72/17	0.71
Hypertension (%)	54	50	0.74
Cardiac failure (%)	8	9	0.95
Ischemic heart disease (%)	8	13	0.55
Diabetes mellitus (%)	17	15	0.87
COPD (%)	17	13	0.68
Neoplasia (%)	91	85	0.41
**Biological data**			
Haemoglobin (g L^-1^)	12 ± 2	13 ± 2	0.12
Haematocrit (%)	37 ± 5	39 ± 4	0.14
Albumin (g L^-1^)	36 ± 4	35 ± 4	0.76
Prealbumin (g L^-1^)	0.25 ± 0.07	0.24 ± 0.06	0.48
Creatinine (μmol L^-1^)	82 ± 31	78 ± 23	0.52
Procalcitonin (mg L^-1^)	0.07 ± 0.04	0.08 ± 0.11	0.76
CRP (mg L^-1^)	6 ± 7	7 ± 16	0.74
Lactate (mmol L^-1^)	1.4 ± 0.6	1.3 ± 0.5	0.48

Data are presented as means ± SD, or absolute values (%).

Abbreviations: ASA, American Society of Anaesthesiology physical status; BMI, body mass index; COPD, chronic obstructive pulmonary disease; CRP, C-reactive protein; P-POSSUM, Portsmouth Physiological and Operative Severity Score for the Enumeration of Mortality and Morbidity.

**Figure 2:**
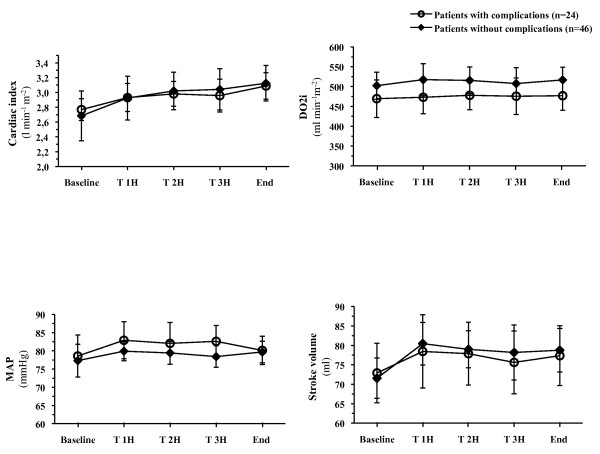
**Cardiac index, oxygen delivery index (DO_2_i), stroke volume and mean arterial pressure (MAP) in patients who did (*n *= 24) and did not (*n *= 46) develop postoperative complications**. There was no difference in any variable between groups at any time point. Data are expressed as means ± 95% CI.

The amounts and types of fluid infused intraoperatively are listed in Table 2. There was no difference in the total volume of fluid infused between groups (*P *= 0.44), although less crystalloids were administered in patients with complications (*P *< 0.01). Additional fluid boluses were also significantly higher in these patients (*P *< 0.01). There was no difference in blood transfusion and in the number of patients who required ephedrine chlorhydrate and dobutamine (Table 2). There were no relevant differences in the principal haemodynamic (Figure 2) and biological variables in patients with and without complications, except for haemoglobin concentration (11.5 ± 1.3 vs. 12.2 ± 1.1 g dL^-1^, *P *= 0.04 at the end of surgery) and excess bases (Table 3). There was also no relevant difference regarding serum lactate concentration: (3.1 ± 2.5 *vs*. 2.3 ± 1.4 mmol L^-1^, *P *= 0.16 and 1.7 ± 0.8 vs. 1.6 ± 0.6 mmol L^-1^, *P *= 0.59 at PACU admission and at postoperative Day 1, respectively) nor in serum creatinine between patients who did and did not develop postoperative complications.

**Table 2:** Intraoperative fluid management in patients with and without postoperative complications

	Patients with complications (*n *= 24)	Patients without complications (*n *= 46)	*P*
Total volume of fluid infused (mL)	4,725 (3,600 to 5,300)	4,525 (3,850 to 6,000)	0.44
Total volume of crystalloids infused (mL)	3,255 (2,760 to 4,300)	4,100 (2,760 to 5,660)	0.04
Total volume of colloids infused (mL)	750 (680 to 1,250)	250 (60 to 500)	< 0.01
Fluid challenge			
No. of challenge per patient	4 ± 2	2 ± 2	< 0.01
No. (%) of patients who needed	21 (87)	34 (74)	0.19
Blood transfusion, N (%) of patients	6 (25%)	7 (15%)	0.31
Urine output (mL)			
Intraoperative	600 (390 to 800)	500 (300 to 975)	0.46
Day 1	1,350 (800 to 1,950)	2,000 (1,350 to 3,100)	0.001
Day 2	2,000 (1,150 to 2,500)	2,450 (1,525 to 3,000)	0.45
Vasoactive support			
Ephedrine chlorhydrate, N (%) of patients	20 (83%)	43 (93%)	0.18
Dobutamine, N (%) of patients	0	1	NR

Data are presented as means ± SD, medians (interquartile range) or absolute values (%). NR, not related.

**Table 3:** Intraoperative biological data

	Patients with complications (*n *= 24)	Patients without complications (*n *= 46)	*P*
**Arterial pH**			
Baseline	7.42 ± 0.03	7.43 ± 0.04	0.27
T 1H	7.39 ± 0.04	7.41 ± 0.04	0.11
T 2H	7.39 ± 0.04	7.40 ± 0.02	0.17
T 3H	7.38 ± 0.05	7.39 ± 0.03	0.78
End of surgery	7.37 ± 0.05	7.38 ± 0.05	0.26
**Arterial PO_2_, mmHg**			
Baseline	186 ± 39	195 ± 52	0.59
T 1H	185 ± 43	180 ± 41	0.56
T 2H	173 ± 44	179 ± 37	0.61
T 3H	172 ± 43	178 ± 35	0.46
End of surgery	178 ± 44	181 ± 37	0.59
**Arterial PCO_2_, mmHg**			
Baseline	36 ± 5	36 ± 4	0.90
T 1H	37 ± 4	36 ± 3	0.41
T 2H	37 ± 4	36 ± 3	0.53
T 3H	36 ± 5	36 ± 3	0.62
End of surgery	36 ± 5	37 ± 3	0.36
**BE, mmol L^-1^**			
Baseline	-1.7 ± 4.3	-0.5 ± 2.6	0.71
T 1H	-3.2 ± 2.7	-1.1 ± 2.2	0.02
T 2H	-2.6 ± 2.9	-1.5 ± 2.1	0.31
T 3H	-2.4 ± 2.8	-2.4 ± 2.2	0.65
End of surgery	-4.0 ± 2.6	-2.8 ± 2.7	0.11
**SaO_2_, %**			
Baseline	98 ± 1.1	99 ± 0.8	0.03
T 1H	98 ± 1.0	99 ± 0.6	0.001
T 2H	98 ± 1.4	98 ± 0.8	0.025
T 3H	98 ± 1.2	98 ± 1.0	0.16
End of surgery	98 ± 0.8	98 ± 0.7	0.21

Data are presented as means ± SD.

BE, base excess; SaO_2_, arterial saturation of oxygen; T, time.

### Association with outcome

**Figure 3:**
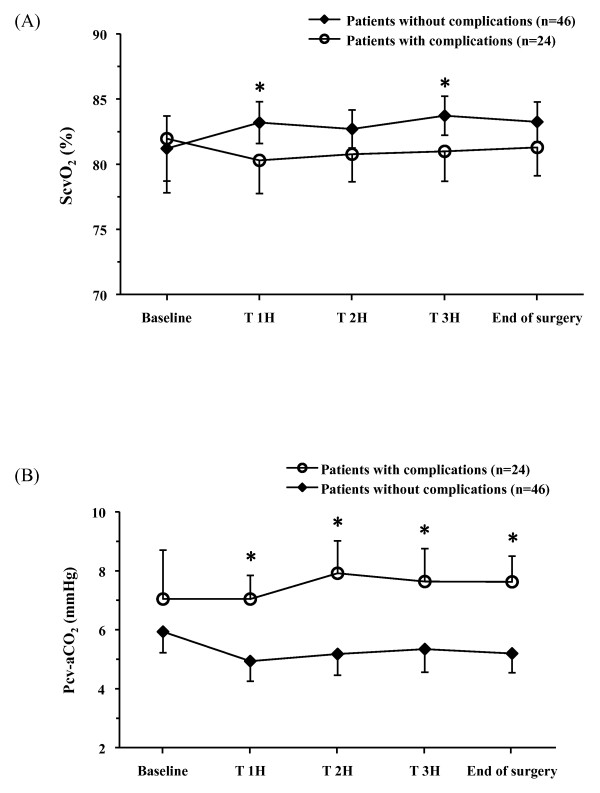
**Trends in ScvO_2 _(a) and P(cv-a)CO_2 _(b) in patients who did (*n *= 24) and did not (*n *= 46) develop postoperative complications**. Data are expressed as means ± 95% CI. * *P *< 0.05.

At baseline there was no difference in ScvO_2 _values between patients who did and did not develop postoperative complications (82 ± 10 vs. 81 ± 9%, respectively, *P *= 0.75) (Figure 3a). Compared with uncomplicated patients, mean ScvO_2 _(78 ± 4 vs. 81 ± 4%, *P *= 0.017) and minScvO_2 _(67 ± 6 vs. 72 ± 6%, *P *= 0.0017) were both lower in patients with complications. Univariate analysis identified four variables associated with postoperative complications: minScvO_2 _(*P *= 0.0028), treatment group (C-GDT and R-GDT, *P *= 0.0067), BMI (*P *= 0.017) and the need for additional fluid bolus (*P *= 0.035). Multivariate analysis showed that the need for additional fluid bolus (OR = 1.46 (95% CI: 1.12 to 2), *P *= 0.005) and minScvO_2 _< 70% (OR = 4.0 (95% CI: 1.23 to 12.5}, *P *= 0.019) were independently associated with postoperative complications. The area under the ROC curve for ScvO_2 _was 0.736 (95 CI%: 0.61 to 0.86) according to the occurrence of postoperative complications. The optimal ScvO_2 _value was 70.6% (sensitivity 72.9%, specificity 71.4%) for discrimination of patients who did and did not develop complications. Intraoperative characteristics of patients with mean ScvO_2 _> 71% who did and did not develop postoperative complications are listed in Table 4.

**Table 4:** Intraoperative haemodynamic data and fluid management in patients with mean ScvO_2 _> 71%

	Patients with complications (*n *= 10)	Patients without complications (*n *= 36)	*P*
CI, L min^-1 ^m^-2^			
Baseline	2.9 ± 0.8	2.7 ± 0.5	0.33
Mean	3.0 ± 0.7	2.9 ± 0.5	0.94
End of surgery	3.2 ± 0.7	3.1 ± 0.6	0.79
DO_2_i, mL min^-1^m^-2^			
Baseline	497 ± 94	510 ± 126	0.73
Mean	500 ± 73	518 ± 108	0.74
End of surgery	502 ± 74	527 ± 113	0.65
SV, mL			
Baseline	75 ± 13	74 ± 19	0.52
Mean	79 ± 10	78 ± 17	0.47
End of surgery	82 ± 14	82 ± 20	0.84
MAP, mmHg			
Baseline	76 ± 14	78 ± 17	0.93
Mean	76 ± 8	79 ± 11	0.81
End of surgery	75 ± 7	79 ± 10	0.37
Total volume of fluid Infused			
Crystalloids, mL	3,375 (2,712 to 4,455)	4,250 (2,700 to 6,000)	0.18
Colloids, mL	5 (500 to 1,188)	250 (0 to 500)	0.11
Blood transfusion, N (%) of patients	2 (20%)	8 (22%)	0.63
Vasoactive support			
Ephedrine chlorhydrate, N (%) of patients	8 (80%)	34 (94%)	0.15
Dobutamine, N (%) of patients	0	1	NR

Data are presented as means ± SD, median (interquartile range) or absolute values (%).

Abbreviations: CI, cardiac index; DO_2_i, oxygen delivery index; MAP, mean arterial pressure; NR, not related; ScvO_2, _central venous oxygen saturation; SV, stroke volume.

### Trends in P(cv-a)CO_2_

At baseline there was no difference in P(cv-a)CO_2 _values between patients with and without complications (*P *= 0.22) (Figure 3b). Mean P(cv-a)CO_2 _was larger in patients who developed complications than in those who did not (7.8 ± 2 vs. 5.6 ± 2 mmHg, *P *< 10^-6^). The area under the ROC curve for P(cv-a)CO_2 _was 0.751 (95% CI: 0.71 to 0.79). The best cut-off P(cv-a)CO_2 _value was 6 mmHg (sensitivity 79%, specificity 66%, positive predictive value 56%, negative predictive value 85%) for discrimination of patients who did and did not develop complications. When we considered P(cv-a)CO_2 _with overall complications (not only those associated with sepsis) in all of the 30 patients, the difference between patients who did and did not develop complications still remained significant. We constructed the ROC curve and found that a P(cv-a)CO_2 _of 6 mmHg predicted the occurrence of complications with 75% sensitivity, 50% specificity, predictive positive value of 0.13 and predictive negative value of 0.95 (AUC 0.648, 95% CI 0.58 to 0.72).

**Figure 4:**
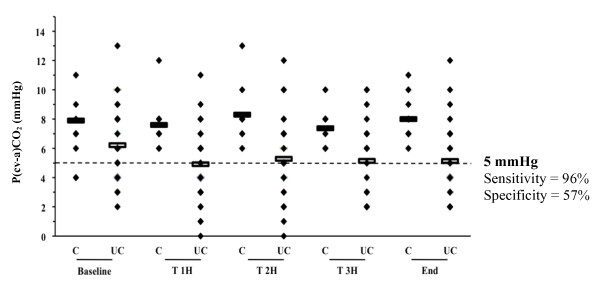
**Individual values of P(cv-a)CO_2 _according to the occurrence of postoperative complications in patients with ScvO_2 _≥71%**. Abbreviations: C, patients with complications; UC, patients without complications.

In patients with ScvO_2_≥71%, mean P(cv-a)CO_2 _was larger in patients who developed postoperative complications than in patients with ScvO_2_≥71% who did not (7.7 ± 2 *vs*. 5 ± 2 mmHg, respectively, *P *< 10^-6^). The area under the ROC curve for P(cv-a)CO_2 _was 0.785 (95% CI: 0.74 to 0.83) with 5 mmHg as the best threshold value (sensitivity 96%, specificity 54%, positive predictive value 41%, negative predictive value 98%) for discrimination of patients with ScvO_2 _≥71% who did and did not develop postoperative complications (Figure 4).

## Discussion

Recently published data clearly demonstrate that low ScvO_2 _during major abdominal surgery is associated with an increased risk of postoperative complications [13-15]. In this study, using Doppler-derived deltaPV as a goal-directed approach, it was observed that high ScvO_2 _(≥71%) did not necessarily preclude postoperative complications. In this context, the presence of a P(cv-a)CO_2 _value > 5 mmHg may be a useful complementary tool to identify patients with ScvO_2_≥71% who might remain insufficiently optimized haemodynamically.

There is growing evidence that individualized fluid loading through goal-directed protocols, titrated by dynamic indices of either flow or preload, improves patient outcome, and is superior to the assessment of standard haemodynamic parameters such as mean arterial pressure (MAP), heart rate or central venous pressure, to prevent inadequate or excessive fluid administration [4,9,33,34]. Although the underlying mechanisms remain controversial, most goal-directed therapy (GDT) protocols include fluid loading, alone or combined with inotropes, to prevent O_2 _debt by maintaining tissue perfusion [3]. In our recently published randomized study of patients treated with an individualized oesophageal Doppler-guided fluid substitution protocol, we found that crystalloid restriction (6 *vs*. 12 mL kg^-1^h^-1^) was associated with increased postoperative complications [17]. Interestingly, the results also indicated that individualized optimization of preload by colloid loading might not have been sufficient to promote optimal tissue perfusion and oxygenation, as indicated by reduced ScvO_2 _values (69 ± 6 vs. 72 ± 6 mmHg, *P *= 0.04) in the restricted-GDT group of patients [17].

Although the prognostic significance of reduced ScvO_2 _and the benefit of its normalization in early goal-directed protocols have been proposed [13,19,35], both normal and high ScvO_2 _values do not preclude microcirculatory failure [19]. In this context, in patients treated with an early GDT-based sepsis resuscitation protocol, Jones and colleagues [36] and Vallee and colleagues [20] showed that either lactate clearance or P(cv-a)CO_2 _might be useful to identify persistent tissue hypoperfusion when the ScvO_2 _goal has been reached with apparent normal DO_2_/VO_2 _ratio. It was also observed that, in surgical patients, an individualized preload-targeted fluid loading to maintain tissue perfusion was not sufficient to prevent significant differences in outcome [17]. Interestingly, mean P(cv-a)CO_2 _was larger in patients with complications with a "normalized" DO_2_/VO_2 _ratio (ScvO_2 _≥71%), than in patients without complications, with 5 mmHg as the best threshold value. According to ScvO_2_, CI and DO_2_i values, enlarged P(cv-a)CO_2 _could be explained by a certainly small but persistent tissue hypoperfusion degree in patients who go on to develop postoperative complications. The increase in venous PCO_2 _would reflect a state of insufficient flow relative to CO_2 _production [37]. This condition has been demonstrated previously [22,38]. Indeed, Vallet and colleagues [22] evidenced that the venous-to-arterial CO_2 _gap (PCO_2 _gap) increased during low blood flow-induced tissue hypoxia (ischemic hypoxia) while it remained unchanged during hypoxemia-induced hypoxia (hypoxic hypoxia).

These results are in agreement with those of Bakker and colleagues [24] who showed that, in patients with septic shock, the PCO_2 _gap was smaller in survivors than in non-survivors, despite quite similar CI, DO_2 _and VO_2 _values. In septic shock patients, characterized by an increased PCO_2 _gap and a low flow state, fluid challenge was found to lower the PCO_2 _gap while increasing cardiac output [39]. In contrast, no significant changes in cardiac output and PCO_2 _gap were found in patients with normal PCO_2_, thus confirming the relationship between an increased PCO_2 _gap and insufficient flow [39]. According to our P(cv-a)CO_2 _values and the associated trends in both lactate and base excess concentrations (Tables 3 and 4), it can be speculated that, despite an optimized preload with fluid challenge, patients with ScvO_2 _values ≥71% who developed complications might have had a relatively insufficient flow state and might have benefited from an increased CI as suggested by the study of Donati [13]. Previous reports have shown that, under conditions where O_2 _demand exceeds O_2 _consumption (VO_2_), ScvO_2 _(and O_2 _extraction) does not accurately reflect the O_2_demand/DO_2 _relationship [40]. According to the modified Fick equation applied to CO_2_, PCO_2 _gap is linearly related to CO_2 _production (VCO_2_) and inversely related to CI [23]. Considering the respiratory quotient (VCO_2_/VO_2 _ratio), VCO_2 _is directly related to O_2 _consumption (VO_2_) [23]. Under conditions of adapted cardiac output to VO_2_, even if the CO_2 _produced is higher than normal because of an additional anaerobic CO_2 _production, in the presence of sufficient flow to wash out the CO_2 _produced by the tissues, the PCO_2 _gap should not be increased [22]. Conversely, low blood flow can result in a widening of the PCO_2 _gap even if no additional CO_2_production occurs because of a CO_2 _stagnation phenomenon [38,41]. The association of these situations may explain, in the current study, the combination of "normal" ScvO_2 _values and increased P(cv-a)CO_2 _values. It can be argued that, despite an apparently normal CI during the entire surgical procedure, this condition could relate to a relatively insufficient flow state, and could be associated with an increased O_2 _demand and hence increased CO_2 _production. Whether increasing in the CI may be beneficial in this situation remains to be evaluated.

These findings may be difficult to generalize because the study has several limitations. First, we are aware that the number of patients included was relatively small which could limit the external validity of the study, and that complementary data are needed to confirm the results. Nevertheless, when we considered that at least one measurement of P(cv-a)CO_2 _> 5 mmHg would represent a risk factor associated with the occurrence of postoperative complications, we found a *post-hoc *power of 52%. Furthermore, when we considered the number of episodes of P(cv-a)CO_2_, we found that more than or equal to three episodes of P(cv-a)CO_2 _> 5 mmHg was associated with a 20% risk of postoperative complications (with a post-hoc power calculation > 90%). Second, while the threshold ScvO_2 _value is very similar to that described previously in a comparable surgical population, the optimal threshold P(cv-a)CO_2 _value of 5 mmHg in line with a 71% ScvO_2 _goal might be subject to criticism. It might be considered that a higher ScvO_2 _(that is, ≥73%) would represent a more appropriate target value [40]. Third, potential confounders such as hypothermia, which may decrease cellular respiration and, therefore, CO_2 _generation [21], might have affected the results. Nevertheless, during the entire surgical procedure, special attention was taken to maintain normothermia. In addition, except for fluid therapy, intraoperative management was similar in the two groups of patients. Although there was a significant difference in the volume of fluids infused, this was not associated with postoperative complications with logistic regression (*P *= 0.16 and *P *= 0.49 for crystalloids and colloids, respectively). Even after adjustment P(cv-a)CO_2 _> 5 mmHg still remains associated with the occurrence of postoperative complications (*P *< 0.001). Fourth, the use of central venous-to-arterial PCO_2 _difference as a surrogate for mixed venous PCO_2 _gap might be a further limitation. Nevertheless, it has recently been found that central venous PCO_2_, obtained from a simple central blood sample instead of a pulmonary arterial blood sample, is a valuable alternative to PvCO_2 _and that correlation with CI still exists in this context [21]. In addition, measurement of P(cv-a)CO_2 _instead of P(v-a)CO_2 _may be more convenient in a surgical context.

## Conclusions

There is strong support today for the use of individualized goal-directed fluid substitution during high-risk surgery. Although ScvO_2 _reflects important changes in the O_2 _delivery/consumption relationship, it is speculated that P(cv-a)CO_2 _might reinforce the value of ScvO_2 _to identify insufficient flow and tissue hypoperfusion during high-risk surgery. In this context, P(cv-a)CO_2 _could be a useful complementary tool to ScvO_2 _to identify patients who remain inadequately managed when the optimization goal has been reached by volume loading during a GDT protocol. Future research is needed to validate this finding.

## Key messages

• Early detection and correction of tissue hypoperfusion were shown to improve outcome during high-risk surgery.

• Central venous-to-arterial CO_2 _difference might serve as a complementary tool to ScvO_2 _to identify insufficient flow when individualized optimization of intravascular status has been reached with fluid loading.

• Larger randomized trials are now required to confirm the benefit of this approach.

## Abbreviations

ASA: American Society of Anaesthesiology; CI: cardiac index; CRP: C-reactive protein; DeltaPV, respiratory variation in peak aortic flow velocity; DO_2_: oxygen delivery; DO_2_i: oxygen delivery index; GDT: goal-directed therapy; MAP: mean arterial pressure; PACU: post-acute care unit; PCT: procalcitonin; P(cv-a)CO_2_: central venous-to-arterial carbon dioxide difference; P-Possum: Portsmouth Physiological and Operative Severity Score for the Enumeration of Mortality and Morbidity; PRBCs: packed red blood cells; P(v-a)CO_2_: mixed venous-to-arterial carbon dioxide difference; ROC: receiver operating characteristic; ScvO_2_: central venous oxygen saturation; SV: stroke volume; SvO_2_: mixed venous oxygen saturation; VO_2_: oxygen consumption.

## Competing interests

The authors declare that they have no competing interests.

## Authors' contributions

EF and JMC conceived and designed the original study. BV suggested complementary analysis (assessment of P(cv-a)CO_2_). MJ and RG were responsible for patient enrolment and participated in data acquisition. EF, ER, BV and JEB drafted the manuscript. All authors read and approved the final manuscript.
